# Bovine Hydroxyapatite-Based Bone Scaffold with Gentamicin Accelerates Vascularization and Remodeling of Bone Defect

**DOI:** 10.1155/2021/5560891

**Published:** 2021-05-05

**Authors:** Aniek S. Budiatin, Maria A. Gani, Samirah, Chrismawan Ardianto, Aulia M. Raharjanti, Indah Septiani, Ni Putu K. P. Putri, Junaidi Khotib

**Affiliations:** ^1^Department of Clinical Pharmacy, Faculty of Pharmacy, Universitas Airlangga, Surabaya, Indonesia; ^2^Doctoral Programme of Pharmaceutical Sciences, Faculty of Pharmacy, Universitas Airlangga, Surabaya, Indonesia

## Abstract

Osteomyelitis is an infectious disease which is also a major complication of bone defects. This study aims to determine the effect of bovine hydroxyapatite-gelatin-based bone implants with gentamicin as an antibiotic (BHA-GEL-GEN implant) on the regeneration of bone defects *in vivo*. The BHA-GEL-GEN and BHA-GEL implants were made by direct compression. *In vivo* study was carried out with Wistar rats. The rats were divided into three groups: negative control, BHA-GEL implant, and BHA-GEL-GEN implants. The defect model used was the burr hole defect model with diameter 2.2 mm and 2 mm deep. After 2, 7, 14, and 28 days, the rats were sacrificed. Bone integrity was carried out using X-ray radiography. Radiological examination was performed using haematoxylin and eosin (HE) staining and immunohistochemical techniques with anti-vascular endothelial growth factor (VEGF) and anti-alkaline phosphatase (ALP) antibodies. Based on the radiograph, the implanted group had accelerated bone growth in the defect area. Semiquantitative data from HE staining showed that the implanted group had accelerated migration of osteoclasts, osteoblasts, and osteocytes in the defect area. The immunoreactive score showed that the BHA-GEL-GEN group had higher VEGF expression compared to two other groups. The three groups did not provide a significant difference in ALP expression. In conclusion, the BHA-GEL-GEN implant causes accelerated bone defects repair by accelerating tissue vascularity and does not interfere with the bone remodeling process. Therefore, the BHA-GEL-GEN implant is potentially a biomedical material for osteomyelitis therapy.

## 1. Introduction

Osteomyelitis is a major complication of various bone pathological conditions, such as fractures and total joint arthroplasty [1]. Chronic osteomyelitis can disturb the quality of life of the patient. Huang et al. [2] reported that chronic osteomyelitis increased patient mortality, especially in elderly patients. Like other infectious conditions, bone infection requires antibiotic therapy to eradicate the infectious pathogen. Besides that, antibiotics also function as prophylaxis to prevent infection in bone defects [1]. However, the use of antibiotics in the bone tissue is challenging. This is because the penetration of the drug into bone and joint tissue is very low. Oral antibiotic therapy, in general, cannot produce sufficient MIC for bone tissue. On the other hand, the parenteral route may be toxic when the MIC in the bone tissue is reached [1, 3]. Because of this, many biomaterials are developed as an antibiotic delivery system for bone tissue, one of which is in the form of implants [1, 3].

Hydroxyapatite is one of the most widely developed biomaterials for bone implants. Hydroxyapatite is a calcium phosphate derivative with chemical formula and physical properties similar to hydroxyapatite found in human bones. Hydroxyapatite can be obtained in several ways. For example, it is synthesized from calcium and phosphate or directly extracted from natural sources such as bovine bone. Hydroxyapatite from bovine bone is called bovine hydroxyapatite (BHA) [4]. One of the differences between BHA and synthetic hydroxyapatite is that BHA has a carbonate group that increases osteoblast proliferation and differentiation, thereby making bone matrix synthesis faster [5, 6]. However, BHA as a single implant material is easily brittle [7]. For this reason, a polymer is needed to increase the mechanical strength of the implant. Gelatin is a denatured polymer from collagen making it similar to the organic component of bone, which is also dominated by collagen protein [8]. Because of this, implants with the BHA-GEL matrix may mimic the natural properties of bone and potentially deliver antibiotics such as gentamicin (GEN) to bone tissue. Based on our previous study, implants with the BHA-GEL matrix with GEN as a therapeutic agent were able to inhibit the growth of *Staphylococcus aureus* linearly [3].

Several literature reviews have shown that some drugs negatively affect bone metabolism [9, 10]. However, there is no enough information about the effect of GEN addition in the BHA-GEL matrix on bone tissue regeneration. Therefore, this study aimed to examine GEN's addition to the implant matrix on the *in vivo* bone regeneration process. Besides functioning as a drug delivery system, the implant is designed to support defective bone tissue, so it is also suggested to accelerate bone regeneration.

## 2. Materials and Methods

### 2.1. Fabrication of BHA-GEL-GEN Implant

Bovine hydroxyapatite (BHA) was extracted from bovine bone at temperature of 1000°C based on the procedure of the previous study [4]. A total of 10 grams of BHA, 5 ml 20 wt% gelatin (GEL, Cartino, Thailand), and 10% gentamicin (GEN, Yantai Justaware Pharmaceutical, China) are mixed until reaching homogeneous mixture to form a mass that can be made as granule. The granules were then immersed in a 0.5 wt% glutaraldehyde (GTA, Merck, USA) for 24 hours. The granules were washed until they were free from GA with distilled water and dried at 40°C in oven for 24 hours. Furthermore, 25 mg of granule was weighed and molded with 0.5 tons of weight to form an implant with a diameter of 2 mm. The GEN dosage used for each implant was 0.041 mg/25 mg.

### 2.2. In Vivo Study

A total of 72 male Wistar rats weighing 200–250 grams adapted for at least one week were used as experimental research animals. Before the operation, the mice were anesthetized with xylazine 8 mg/KgBW and ketamine 25 mg/KgBW and were given ampicillin at dose of 25 mg/KgBW intramuscularly to prevent infection. After that, the hair in the femur area was shaved and disinfected with 70% alcohol. After that, an incision is made in the femur until the bone is visible. Then, drilling was carried out on the distal part of the femur with a defect of 2.2 mm in diameter and a depth of about 2 mm. The treatment in mice was divided into three groups: the first group was the negative control group, which is a defect model without implant, the second group was implanted with BHA-GEL implant, and the third group was implanted with BHA-GEN-GEN implant. After the operation is complete, the wound is sutured and cleaned with gauze moistened with normal saline solution followed by betadine, bandaged with gauze, and then plastered. Wound care is carried out every day by smearing the wound with betadine and changing the plaster.

On days 2, 7, 14, and 28 after surgery, the rats were terminated by lethal doses of ketamine and xylazine (3 times the anaesthetic dose). Furthermore, the femur was taken and put into a container containing 10 w/v% formalin solution at room temperature. Bone decalcification is carried out by immersing the bone in 10 w/v% EDTA solution until it becomes soft.

### 2.3. Radiology Examination

Evaluation of bone integrity was performed using X-ray radiography and clarified with ImageJ 1.52a (Wayne Rasband National Institutes of Health, USA).

### 2.4. Haematoxylin and Eosin Staining

The histological examination started with processing the paraffin blocks by dehydrating with serial alcohol (70% to 100%) for 60 minutes each. Subsequently, the clearing was performed with xylol three times for 15 minutes each. After that, the infiltration process was carried out with paraffin liquid for three times of transfer for 60 minutes each in an incubator at 60°C. The tissue is then implanted in paraffin liquid and cooled at room temperature. Each paraffin block was then cut using a rotary microtome with a thickness of 4-6 *μ*.

The cell morphology was stained with haematoxylin and eosin dye. The slide is dipped in xylol three times for 5 minutes each and was hydrated with alcohol (96% to 70% alcohol) for 2 minutes each. The slides were then rinsed using running water for 10 minutes, put in Mayer's haematoxylin for 15 minutes, rinsed under running water, and checked under a microscope. Furthermore, the slide was put in 1% eosin solution for 30 seconds, dehydrated, and cleared, followed by mounting with the EZ mount.

### 2.5. Immunohistochemistry

The immunohistochemical technique was used to stain VEGF and ALP immunopositive cells. The slide was deparaffinised with xylol three times for 5 minutes each, followed by rehydration with alcohol (absolute alcohol to 70%) for 4 minutes each, and washed with running water for 5 minutes. After that, the slide was blocked with 0.5% endogenous peroxide for 5 minutes and washed with running water for 5 minutes. After that, antigen retrieval decloaking chamber was performed, cooled for 20 minutes, and washed with PBS for 3 minutes. After that, sniper blocking was done for 15 minutes. Then, the slide was incubated with rabbit anti-rat VEGF primer (cat. no. PA1-21796, Thermo Fisher Scientific, 1 : 100 dilution) or rabbit anti-rat ALP (cat. no. PAB091Ra01, USCN Life, 1 : 200 dilution) and washed in PBS for 3 minutes. After that, the universal linked was done for 20 minutes, and slide was washed in PBS for 3 minutes. Then, Trekavidin-HRP Label was carried out for 10 minutes and washed in PBS for 3 minutes. After that, the slide was reacted with Chromogen DAB + Buffer Substrate for 2 to 5 minutes, followed by rinsing under running water for 5 minutes. Then, the slide was counterstained with haematoxylin for 1 to 2 minutes, followed by rinsing (twice) under running water for 5 minutes each. After that, the slide was dehydrated with alcohol (70% to absolute alcohol) for 5 minutes each, followed by clearing with xylol three times for 5 minutes each. Finally, mounting was carried out on the slide (ecomount) and covered with a cover glass.

## 3. Results

**Figure 1:**
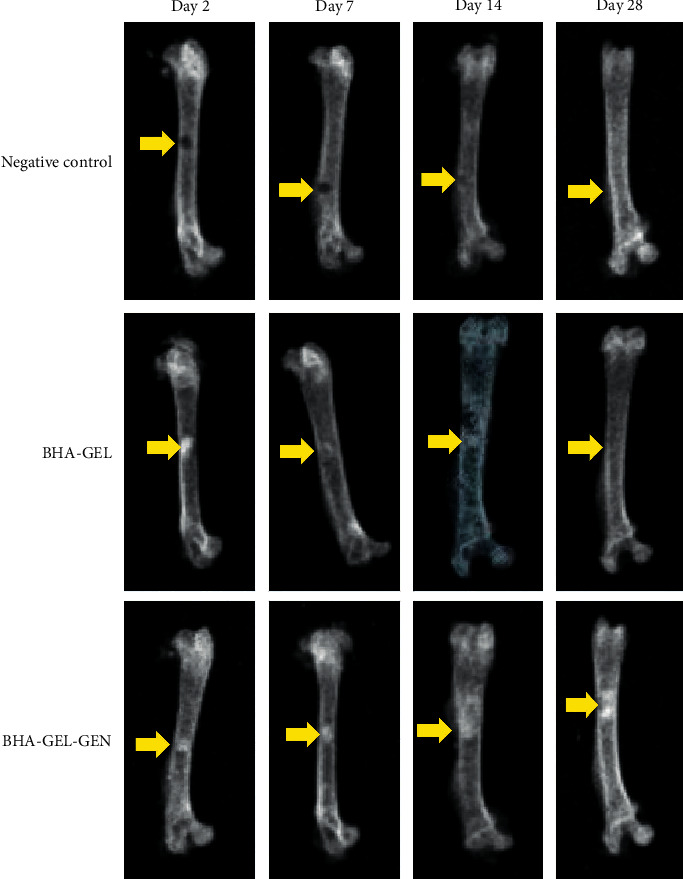
Radiograph of rat femur. The yellow arrow indicates the location of the defect. The implanted group showed accelerated bone growth in the defect area.

**Figure 2:**
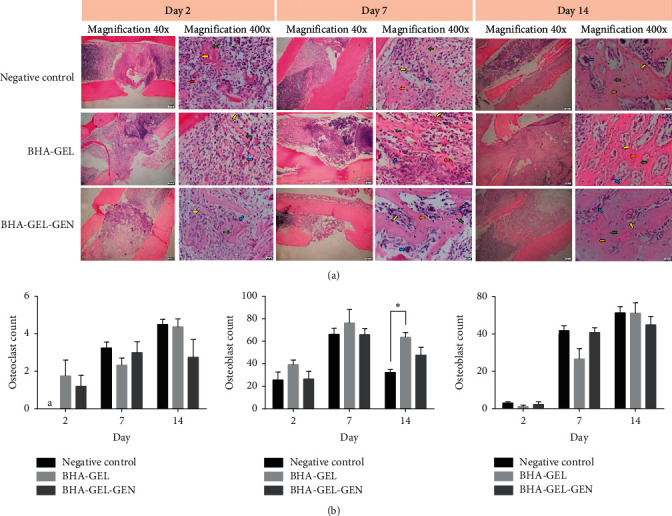
(a) Tangential longitudinal section of rat's femur stained with haematoxylin and eosin. Osteoclasts (blue arrows), osteoblasts (yellow arrows), osteocytes (green arrows), osteoprogenitor cells (red arrows), and cartilage (orange arrows). (b) The number of osteoclasts, osteoblasts, and osteocytes in each group. Each bar represents the mean cell count ± SEM of the two rats observed in at least two different visual fields. ^*∗*^*p* < 0.01 with Kruskal Wallis. ^a^No cells found.

Study about the effect of biomaterials on bone defect repair has been carried out with a burr hole defect model in Wistar rats. After several time points, radiological and histological examinations were performed to examine the bone growth in three groups (negative control, BHA-GEL, and BHA-GEL-GEN). The radiograph showed that the implanted groups experienced accelerated bone growth in the defect area compared to the group without implant (Figure 1). Furthermore, cell morphology present in bone tissue showed that the implanted groups experienced accelerated cell migration; these cells were osteoclasts, osteoblasts, and osteocytes (Figure 2(a)). Based on cell morphology semiquantitative data, the BHA-GEL and BHA-GEL-GEN groups did not show significant differences in the number of osteoclasts, osteoblasts, and osteocytes (Figure 2(b)). So it can be concluded that the addition of gentamicin in the implant formula does not disturb the bone remodeling process.

**Figure 3:**
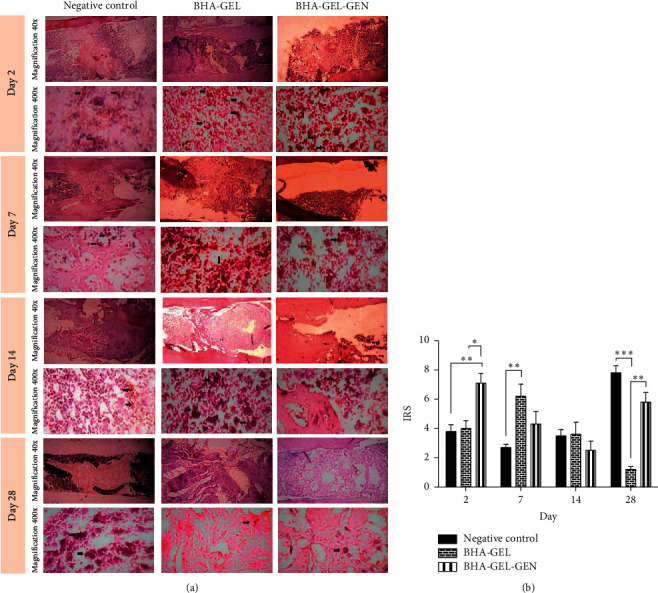
(a) Tangential longitudinal section of rat's femur stained with anti-VEGF. Osteoblasts expressing VEGF (black arrow). (b) Immunoreactive score (IRS) of VEGF in each group. Each bar represents the mean IRS ± SEM of the two rats observed in at least two different visual fields. ^*∗*^*P*=0.05, ^*∗∗*^*p* < 0.05, ^*∗∗∗*^*p* < 0.001 with Kruskal Wallis.

Histological examination with anti-VEGF was carried out to determine the vascularization in the bone tissue of each animal group (Figure 3(a)). On the 2nd postoperative day, the group given BHA-GEL-GEN showed a higher IRS score than the other two groups (One-Way ANOVA, *p*=0.05 vs BHA-GEL, *p* < 0.05 vs negative control; Figure 3(b)). Furthermore, on the 7th postoperative day, the BHA-GEL group showed a higher IRS score than the negative control group (One-Way ANOVA, *p* < 0.05; Figure 3(b)). As for day 28, the negative control group had higher IRS scores than BHA-GEL group (One-Way ANOVA, *p* < 0.001; Figure 3(b)), while the BHA-GEL-GEN group had higher IRS score compared to BHA-GEL group (One-Way ANOVA, *p* < 0.05; Figure 3(b)).

**Figure 4:**
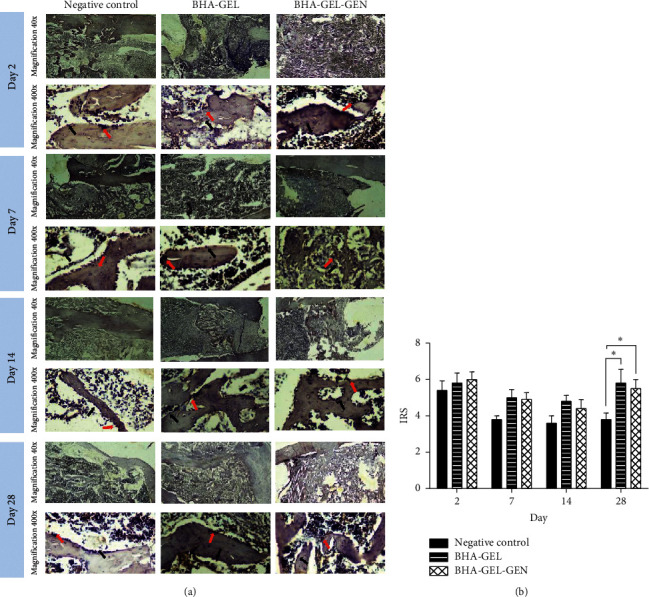
(a) Tangential longitudinal section of rat's femur stained with anti-ALP. Osteoblasts expressing ALP (red arrow), osteoblasts that did not express ALP (black arrow). (b) Immunoreactive score (IRS) of ALP in each group. Each bar represents the mean IRS ± SEM of the two rats observed in at least two different visual fields. ^*∗*^*p* < 0.05 with Kruskal Wallis.

Furthermore, an examination was performed by reacting bone tissue with anti-ALP to examine osteoblast activity in each group (Figure 4(a)). Based on semiquantitative data at days 2, 7, and 14, the two implanted groups did not show any significant difference in IRS score of ALP compared to negative control group (One-Way ANOVA, *p* > 0.05; Figure 4(b)). On the other hand, at day 28, the BHA-GEL and BHA-GEL-GEN groups had higher IRS scores than the negative control group (One-Way ANOVA, *p* < 0.05; Figure 4(b)).

## 4. Discussion

*In vivo* study of the BHA-GEL-GEN implant was carried out on Wistar rats. Based on radiological results, the implanted group, both BHA-GEL and BHA-GEL-GEN, showed accelerated repair of bone defects. This also proofs in histological staining with haematoxylin-eosin In this study, BHA is the main component used as the implant matrix. Based on the previous other study, rabbit implanted with BHA showed accelerated bone growth based on the percentage of new bone and bone-to-material contact compared to other groups [11, 12]. Apart from BHA, another material used as implant matrix is gelatin (GEL). GEL is known to stimulate the proliferation and differentiation of cells involved in bone regeneration [13]. So it can be concluded that the acceleration of bone growth in the defect area is due to the osteoconductive properties of implant matrix.

Bone healing is a complex process that involves a series of cellular and molecular actions. In this process, bone cells interact with other cells to promote tissue healing [14, 15]. Blood vessels play a vital role in maintaining cell viability by providing perfusion to the defect. Blood vessels deliver minerals and growth factors to defective tissue and release paracrine signals that modulate the growth, differentiation, and regeneration of bone cells [15]. Vascular endothelial growth factor (VEGF) is a protein that plays an important role in endothelial proliferation, migration, and activation. VEGF is widely used as a marker of bone tissue vascularization [16]. It is proven that VEGF gene expression and VEGF protein levels also affect bone growth due to biomaterials [17]. In this study, the BHA-GEL-GEN group experienced accelerated vascularization compared to the other two groups based on VEGF expression. This may be due to two factors; acceleration of the inflammatory phase in bone tissue after injury or/and the intact effect of gentamicin on bone tissue. The 3rd day after injury is the inflammatory phase [18]. Umeki [19] found that gentamicin has anti-inflammatory effect by inhibiting the activation of NADPH oxidase in neutrophils. On the other hand, the high expression of VEGF in the BHA-GEL-GEN group may also be due to the intact effect of gentamicin. Jaber and Abdelnoor [20] found that intraperitoneal administration of gentamicin led to an increase in VEGF levels which then supported angiogenesis in C57BL/6 mice. Moreover, the BHA-GEL group also experienced accelerated vascularization than the negative control group. This is possibly because BHA-GEL as the main material for the matrix resembles the inorganic and organic components of bone. Because of this, the implants may also function as bone tissue scaffold and make the growth of blood vessels in the damaged tissue faster than the negative control group [4, 11, 12].

Alkaline phosphatase (ALP) is an enzyme that is produced in the differentiation process of osteoblasts. ALP expression is significantly high at the initial stage of osteoblast differentiation and will decrease when other markers (such as osteocalcin) increase [21]. The present study found that animal group implanted with the biomaterials caused higher ALP expression than the nonimplanted group in the remodeling phase. As previously explained, the main material used as implant's matrix is BHA. BHA is natural hydroxyapatite extracted from bovine bone. BHA has carbonate group as naturally present in human bone hydroxyapatite, and this is not found in synthetic hydroxyapatite [22]. Carbonated hydroxyapatite is known to improve protein adsorption, as well as increased adhesion, proliferation, and osteogenic differentiation of mesenchymal stem cells [23, 24]. Besides, carbonated hydroxyapatite is also known to increase osteoblast proliferation and increase ALP levels *in vitro* [5, 6]. The present study showed that ALP expression in the BHA-GEL and BHA-GEL-GEN groups is not significantly different. This means that the addition of gentamicin to the BHA-GEL implant does not disturb the ongoing bone remodeling process.

## 5. Conclusions

The *in vivo* study of the BHA-GEL-GEN implant was carried out. The BHA-GEL-GEN implants caused accelerated bone tissue vascularization and did not disturb the bone tissue remodeling process, thus causing accelerated repair of bone defects. Therefore, the BHA-GEL-GEN implant will potentially be used to treat osteomyelitis. However, further *in vivo* study with bone infection model is needed.

## Data Availability

All the data used are included in the article.
